# '*Candidatus* Ornithobacterium hominis': insights gained from draft genomes obtained from nasopharyngeal swabs

**DOI:** 10.1099/mgen.0.000247

**Published:** 2019-02-05

**Authors:** Susannah J. Salter, Paul Scott, Andrew J. Page, Alan Tracey, Marcus C. de Goffau, Claire Cormie, Bernardo Ochoa-Montaño, Clare L. Ling, Jiraporn Tangmanakit, Paul Turner, Julian Parkhill

**Affiliations:** ^1^​ Pathogen Genomics, Wellcome Sanger Institute, Hinxton, UK; ^2^​ Department of Biochemistry, University of Cambridge, Cambridge, UK; ^3^​ Shoklo Malaria Research Unit, Mahidol-Oxford Tropical Medicine Research Unit, Faculty of Tropical Medicine, Mahidol University, Mae Sot, Thailand; ^4^​ Centre for Tropical Medicine and Global Health, Nuffield Department of Medicine, University of Oxford, Oxford, UK; ^5^​ Cambodia-Oxford Medical Research Unit, Angkor Hospital for Children, Siem Reap, Cambodia; ^†^​ Present address: Quadram Institute Bioscience, Norwich, UK.; ^‡^​ Present address: Illumina Cambridge Ltd, Little Chesterford, UK.

**Keywords:** *Ornithobacterium*, respiratory tract microbiome, *Pasteurella* mitogenic toxin

## Abstract

‘*Candidatus* Ornithobacterium hominis’ represents a new member of the *Flavobacteriaceae* detected in 16S rRNA gene surveys of people from South-East Asia, Africa and Australia. It frequently colonizes the infant nasopharynx at high proportional abundance, and we demonstrate its presence in 42 % of nasopharyngeal swabs from 12-month-old children in the Maela refugee camp in Thailand. The species, a Gram-negative bacillus, has not yet been cultured, but the cells can be identified in mixed samples by fluorescent hybridization. Here, we report seven genomes assembled from metagenomic data, two to improved draft standard. The genomes are approximately 1.9 Mb, sharing 62 % average amino acid identity with the only other member of the genus, the bird pathogen *Ornithobacterium rhinotracheale*. The draft genomes encode multiple antibiotic-resistance genes, competition factors, *Flavobacterium johnsoniae-*like gliding motility genes and a homologue of the *Pasteurella multocida* mitogenic toxin. Intra- and inter-host genome comparison suggests that colonization with this bacterium is both persistent and strain exclusive.

## Data Summary

The Illumina MiSeq read data are deposited in the EMBL European Nucleotide Archive (ENA) under project accession number ERP107699 (https://www.ebi.ac.uk/ena/data/view/PRJEB25749) [Data Citation 1]. Assembled contigs and their annotations for draft genome OH-22767 are available from the National Center for Biotechnology Information (NCBI) GenBank under accession number UNSC01000000 (https://www.ncbi.nlm.nih.gov/nuccore/UNSC01000000) [Data Citation 2], and OH-22803 under accession number UNSD01000000 (https://www.ncbi.nlm.nih.gov/nuccore/UNSD01000000) [Data Citation 3]. Reference sequences for the full length 16S rRNA gene are available from GenBank under accession numbers LS992475 (https://www.ncbi.nlm.nih.gov/nuccore/LS992475) [Data Citation 4] and LS992476 (https://www.ncbi.nlm.nih.gov/nuccore/LS992476) [Data Citation 5].

**Impact Statement**

The nasopharynx is part of the respiratory tract and hosts a unique microbial community that is established during infancy and changes throughout life. The nasopharyngeal microbiome is important as it includes bacteria that can cause diseases such as otitis media or pneumonia, as well as non-pathogenic species. In Maela, a refugee camp in Thailand, we identified a prevalent bacterial species colonizing children under the age of 2 years and occasionally their mothers. We were not able to culture it from frozen samples, but could visualize the cells microscopically using a fluorescent probe. Its genetic signature can be seen in published data from several countries, suggesting that the species is globally distributed. From analysis of the genome, we confirm it is highly divergent from its closest characterized relative, the respiratory pathogen *Ornithobacterium rhinotracheale,* which infects turkeys, chickens and other birds. We propose the name ‘*Candidatus* Ornithobacterium hominis’ and describe a screening protocol to detect its presence in samples.

## Introduction

During previous work on the nasopharyngeal microbiota of children in the Maela refugee camp in Thailand, an abundant unclassified taxon was discovered through 16S rRNA gene sequencing [1]. It was >99 % identical to other unclassified sequences reported in nasopharyngeal samples from the Gambia [2, 3], Kenya [4] and Australia [5], and the gene shared 93 % nucleotide identity with that of the avian respiratory pathogen *Ornithobacterium rhinotracheale*. On the basis of 16S rRNA gene similarity, the taxon was presumed to represent a new species of *Flavobacteriaceae*, closely related to the genus *Ornithobacterium.* The taxon was of interest because it was ubiquitous in the study group of 21 children, appearing to be a persistent colonizer and at a proportional abundance up to 71 %. The 16S rRNA gene sequences could be divided into three oligotypes [6]: each appeared to be carried persistently and exclusively by their host [1].

As the bacterium could not be cultured from archived swabs, ten DNA samples from the initial study were selected for metagenomic sequencing to maximise recovery of the genome of interest while representing a range of children, ages and 16S rRNA gene oligotypes: seven were successfully sequenced following multiple displacement amplification (MDA). The extracted genomes were then used to design a PCR-based prevalence screen for samples from the Maela cohort and a fluorescent probe to visualize the cells in mixed samples. On the basis of this genomic analysis, we propose the unclassified taxon as ‘*Candidatus* Ornithobacterium hominis’.

## Methods

### Samples

Between 2007 and 2010, a cohort of 955 infants born in the Maela refugee camp on the Thailand–Myanmar border were followed from birth until 24 months of age in a study of pneumococcal colonization and pneumonia epidemiology [7, 8]. Pernasal swabs of the posterior wall of the nasopharynx were collected from each infant at monthly intervals using Dacron tipped swabs (Medical Wire and Equipment). Immediately following collection, the nasopharyngeal swab (NPS) samples were placed into STGG (skimmed milk, tryptone, glucose and glycerol) storage medium and frozen at −80 °C within 8 h. An additional sample was taken if the infant was diagnosed with pneumonia according to World Health Organization clinical criteria [9].

### DNA extraction and sequencing

Preliminary work was performed on DNA extracted from swabs with a FastDNA spin kit for soil (MP Biomedicals), which was then amplified using MDA. MDA reagents were filtered at 0.2 µm, endonuclease digested with φ29 enzyme and UV irradiated (254 nm) prior to use to remove any exogenous DNA from the subsequent amplification reaction [10]. A 3 µl sample was heat denatured with 2 µl heat denaturation buffer [20 mM Tris HCl pH 8.0, 2 mM EDTA and 400 µM PTO random hexamers (Eurofins Genomics)] at 95 °C for 3 min. Reaction master mix (15 µl) [1× RepliPHI reaction buffer, RepliPHI φ29 enzyme (6.7 U µl^−1^) (Epicentre), 0.5 mM dNTP, 50 µM PTO random hexamers, 5 % DMSO, 10 mM DTT] was then added, samples were incubated at 30 °C for 16 h, and the reaction halted by final incubation at 65 °C for 20 min. Eight separate reaction volumes were processed per sample, these parallel reactions were pooled before sequencing using the MiSeq 250 bp paired-end protocol with a 450 bp library fragment size.

### Genome analysis

Samples were selected to maximise recovery of the genome of interest by choosing those with a high proportional abundance of the bacterium from previous 16S rRNA gene sequencing data [1]. Raw reads from MDA samples were first classified using Kraken v. 0.10.6 [11] and parsed to remove any reads classified as mammal, *Moraxella, Haemophilus* or *Streptococcus*. The remaining reads were then assembled using SPAdes v. 3.10.0 [12]. Contigs shorter than 500 bp or with mean coverage below 4× were discarded. For the two well-assembled samples, a blast+ v. 2.7.0 [13] screen of all contigs against the nr database was used to discard those that closely matched other known nasopharyngeal bacteria. The contigs first brought forward for the draft genomes were those that had consistent, low-identity matches to *O. rhinotracheale*. Samples were then reciprocally compared using blast + to find further contigs present in all runs. These curated contig sets were manually improved using Gap5 v. 1.2.14 [14] and targeted PCR for gap closure, resulting in two syntenic draft genomes. The other assemblies with large numbers of short contigs were screened by comparison against the draft genomes using blast+ and extracting all contigs with >10 % length hit. Automated annotation of curated contigs was performed using Prokka v. 1.11 [15] and the RefSeq database [16]. Average nucleotide identity (ANI) and average amino acid identity (AAI) were calculated using the enveomics calculators [17, 18]. ANI used a minimum length 700 bp and minimum identity 70 %, with a 1000 bp fragment window size and 200 bp step. AAI used a 20 % identity cut-off. The reciprocal percentage of conserved proteins (POCP) was calculated as described by Qin *et al.* [19]. The core and accessory genomes were calculated using Roary v. 3.11.3 [20]. Phylogenetic trees were built using RAxML v. 8.2.8 [21]. Metabolic analysis was performed using kegg Mapper v.3.1 [22]: annotation with BlastKOALA [23] and the prokaryotic kegg genes database, followed by comparison of pathways from Reconstruct Module.

### Prevalence screen

Prevalence of carriage in the infant population of Maela was estimated using a quantitative PCR (qPCR) screen direct from NPS storage medium. The 12 months of routine samples from 100 randomly selected infants (excluding twins) were used. The age of child at time of sampling ranged from 359 to 377 days, median 365 days. Concurrent swabs were also acquired from the mothers and screened to assess maternal carriage in relation to infant carriage. qPCR was performed on an Applied Biosystems 7500 RT PCR machine using PowerUp SYBR Green master mix (Applied Biosystems) in 20 µl reaction volumes. The 16S rRNA gene screen targets the V2–V5 region with forward primer 5′-CTTATCGGGAGGATAGCCCG-3′ and reverse 5′-GAAGTTCTTCACCCCGAAAACG-3′, yielding a 700 bp product under the conditions: 94 °C for 5 min (cell lysis); then 40 cycles of 94 °C for 30 s, 53 °C for 30 s, 68 °C for 1 min; ending with a melt curve. A positive result was a cycle threshold (*C*_t_) <40 and peak melting temperature (*T*_m_) of 80–86 °C. The ToxA gene screen with forward primer 5′-TATCTCTCACAGAGCTAGGCTTGAGCGTGG-3′ and reverse 5′-TGCTATATTTGGGAAAGGCGCATGAATACC-3′ yields a 1.95 kb product under the conditions: 94 °C for 5 min (cell lysis); then 40 cycles of 94 °C for 30 s, 58 °C for 2.5 min, 68 °C for 2.5 min; ending with a melt curve. A positive result was *C*_t_ <40 and peak *T*_m_ of 77–79 °C.

A positive result for both targets was interpreted as carriage-positive, a negative result for both targets was interpreted as carriage-negative. Non-concordant results (positive/negative or negative/positive) were treated as a separate group. This assessment of carriage prevalence may be affected by several factors: recent antibiotic consumption, low microbial biomass, age under 6 months (as inferred from previous work [1]) or technical error during swab collection may lead to a lower estimate. Presence of dead bacterial cells in the nasopharynx or cross-contamination during sample handling may lead to false positives. The sample size of 100 was selected as adequate to encompass a predicted prevalence of 10–90 % with a precision of 5% and 95 % confidence interval (CI) [24]. This sample size is approximately one tenth of the total population being estimated, i.e. all 12-month-old children who were born in Maela between 2007 and 2008.

### Protein modelling

The ‘*Candidatus* O. hominis’ ToxA protein sequence was searched using the program fugue [25] against a database of all chains of the Protein Data Bank (PDB) as of June 2017 [26]. Significant similarity with 30 % sequence identity was found for residues 554–1269 to chain X of PDB ID 2EBF [27], with corresponds to the C-terminal region of the *Pasteurella* mitogenic toxin. The matched region was aligned to the chain sequence using fugue, and models were generated with modeller v.9.15 using ‘very slow’ refinement [28]. Visualization of the resulting models was performed on PyMOL Molecular Graphics System v1.8.

### Microscopy

A suspension of NPS STGG sample was fixed overnight at 4 °C in 3 % paraformaldehyde, and dehydrated in suspension with 96 % ethanol. Fluorescent hybridization with an Alexa546-labelled probe (Invitrogen) was performed on a fixed sample in buffered suspension (20 mM Tris-HCl, 0.9 M NaCl, 0.1 % SDS) for 2 h at 55 °C, and washed with 20 mM Tris-HCl, 0.9M NaCl for 5 min at 55 °C. The samples were then suspended in water and applied to standard microscope slides, dried, then incubated in a 300 nM solution of DAPI (4’,6-diamidino-2-phenylindole dihydrochloride) in 1x PBS, for 5 min at room temperature. The slides were rinsed with 1x PBS, rinsed again with water, dried and a coverslip applied with ProLong diamond antifade mountant (Invitrogen). The probe with sequence 5′-GUUCUUCACCCCGAAAACG-3′ targets the V5 region of the 16S ribosomal RNA, corresponding approximately to position 822–840 of the 16S rRNA gene of *Escherichia coli*. It was not found to bind to an *O. rhinotracheale* sample that was fixed and processed in parallel. A Leica TCS SP8 confocal fluorescence microscope was used to visualize probed cells with laser wavelengths 405 nm and 552 nm. Images were captured and processed via Leica Application Suite X software.

### Culture methods

NPS STGG samples were streaked out on 4 % horse blood or chocolate agar (Oxoid blood agar base no. 2; Thermo Fisher Scientific) and incubated at 37 °C in aerobic, enriched CO_2_ or anaerobic conditions for 48 h. Brain heart infusion broth (25 ml) (Thermo Fisher Scientific), with and without 1 µg ampicillin ml^−1^, was inoculated with 5 µl STGG medium and incubated either static or shaking at 37 °C for up to 1 week. Other nasopharyngeal species were recovered from NPS STGG samples following these methods, but ‘*Candidatus* O. hominis’ was not. The *O. rhinotracheale* sample used as a negative control for fluorescent hybridization was cultivated on 4 % horse blood agar in microaerobic conditions at 37 °C for 48 h, colonies were then scraped from plates and fixed using the protocol described above.

## Results

Genomes of ‘*Candidatus* O. hominis’ were assembled from metagenomic data generated on an Illumina MiSeq [Data Citation 1]. Despite significant loss of sequence coverage to human and other bacterial genomes, two samples assembled into 9 and 15 contigs from ‘*Candidatus* O. hominis’, yielding draft genomes predicted to be nearly complete based on the detection of all ribosomal protein genes and by inter-sample comparison. A further five samples assembled into larger numbers of small contigs, which were aligned to the draft genomes and found to cover most of the expected length (Table 1). The ‘*Candidatus* O. hominis’ genome is approximately 1.9 Mb, 20 % smaller than its closest relative *O. rhinotracheale*.

**Table 1.** Sample information Metadata associated with sequenced samples; well-assembled draft genomes are highlighted in green.

Child	Age (months)	Sample	Date	Genome ID (accession no.)	No. of extracted contigs	Total length	Max contig length	Predicted oligotype and proportion*
**ARI0073**	11	08B08946	Nov 2008	OH-22797 (ERS2321173)	302	1.81 Mb	32 kb	OTP-C: 56 %
**ARI0073**	15	09B02844	Mar 2009	OH-22872 (ERS2321176)	41	1.93 Mb	509 kb	OTP-C: 38 %
**ARI0073**	21	09B09452	Sep 2009	OH-22767 (ERS2321172)	15	1.93 Mb	611 kb	OTP-C: 31 %
**ARI0106**	20	09B08231	Aug 2009	OH-22298 (ERS2321170)	183	1.80 Mb	68 kb	OTP-A: 67 %
**ARI0218**	14	09B00559	Jan 2009	OH-22819 (ERS2321175)	172	1.42 Mb	51 kb	OTP-A: 49 %
**ARI0218**	19	09B06140	Jun 2009	OH-22803 (ERS2321174)	9	1.87 Mb	684 kb	OTP-A: 62 %
**ARI0484**	15	09B08221	Aug 2009	OH-22763 (ERS2321171)	205	1.88 Mb	57 kb	OTP-B: 51 %

*16S rRNA gene oligotype and proportional abundance of ‘*Candidatus* O. hominis’ in sample from previous study [1].

### Prevalence

A *Candidatus* O. hominis’ specific real-time PCR detection protocol for V2–V5 of the 16S rRNA gene was designed using full-length gene sequences and tested on NPS STGG samples and on metagenomic DNA of known bacterial composition. This PCR screen was then applied directly to STGG medium from the archived NPS samples of 100 randomly selected 12-month-old infants in Maela, and concurrent swabs from their mothers. A second PCR screen was developed targeting the toxin gene *toxA* and was also performed on the archived NPS samples. The two PCR targets were concordant in infant samples, resulting in 42 positive and 58 negative results, giving an estimated carriage prevalence among 12-month-old infants in Maela of 42 % (95 % CI: 32.3–51.7). From the mothers, 2 samples were positive, 93 negative and 5 were either equivocal or nonconcordant (Table 2). The *C*_t_ values were higher in maternal samples than infants, which may indicate a lower bacterial load. Of the 100 infant samples, 12 were also sequenced during the earlier 16S rRNA gene study [1] and 11 of those qPCR results were in agreement with the previous data. One sample with a predicted proportional abundance of 3 % ‘*Candidatus* O. hominis’ had a negative qPCR result, while two others of low proportional abundance gave positive qPCR results with *C*_t_ values approaching the limit of detection.

**Table 2.** *Candidatus* O. hominis’ prevalence Results of the qPCR screen for two targets in mother and infant samples.

Infant or mother	No. of samples	*‘Candidatus* O. hominis’ PCR	
		16S target	Toxin target
Infant	42	+	+
	58	−	−
Total	100		
Mother	2	+	+
	93	−	−
	5	+/equivocal	−
Total	100		

### Genetic similarity between ‘*Candidatus* O. hominis’ and *O. rhinotracheale*

**Fig. 1.**
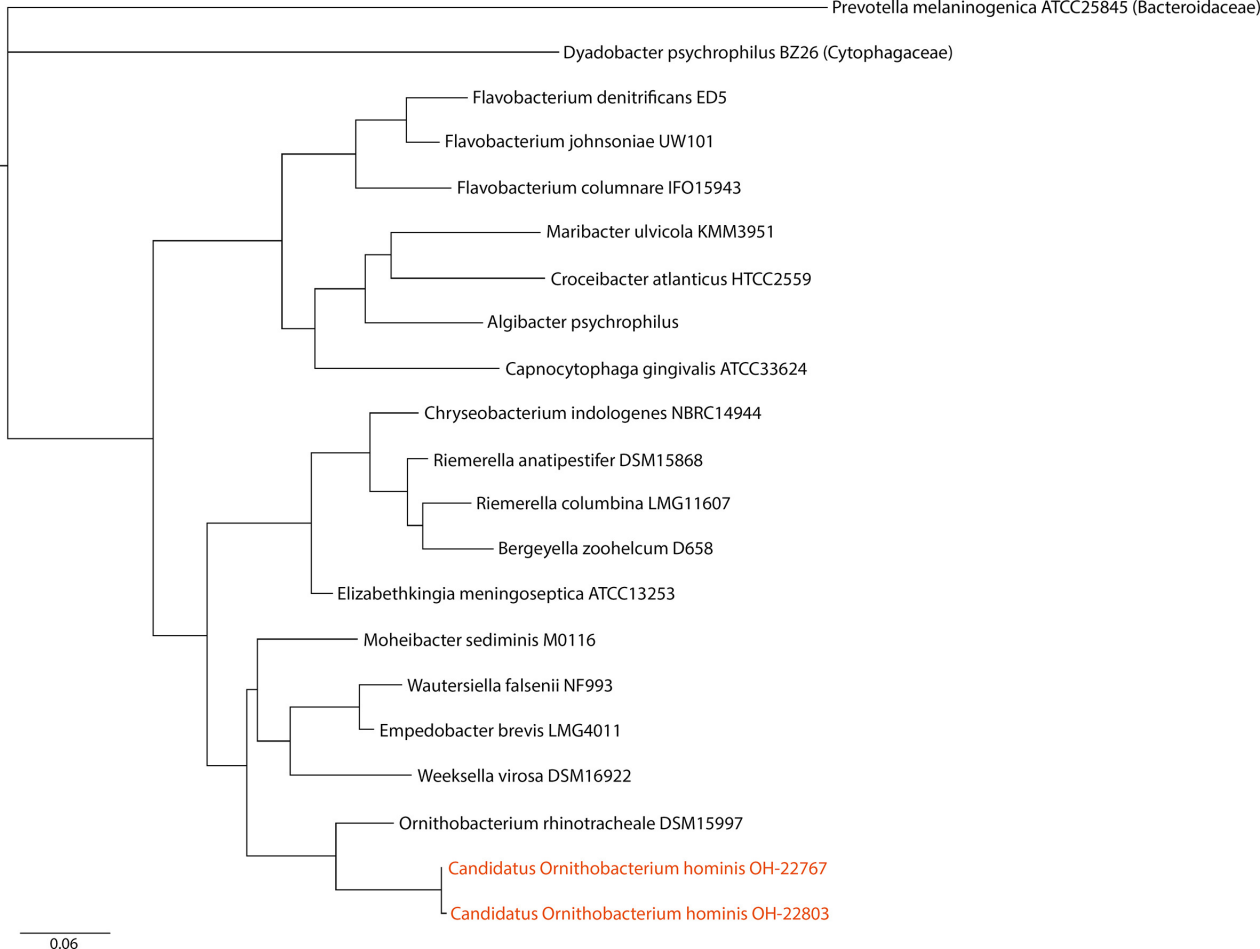
*Candidatus* O. hominis’ in the context of the family *Flavobacteriace*ae. RAxML phylogenetic tree built from 16S rRNA gene sequences (spanning regions V1–V9) from ‘*Candidatus* O. hominis’ and other *Flavobacteriaceae* species, with two members of other families in the phylum. Scale bar indicates mean nucleotide substitutions per site.

The position of ‘*Candidatus* O. hominis’ in the context of the *Flavobacteriac*eae, based on 16S rRNA gene sequences, is illustrated in Fig. 1. The ‘*Candidatus* O. hominis’ draft genomes [Data Citation 2 and 3] appear to be very distantly related to *O. rhinotracheale* by several measures, summarized in Table 3. The ANI between ‘*Candidatus* O. hominis’ and *O. rhinotracheale* UMN-88 [29] [Data Citation 6] can only be calculated from a small fraction of the genome. The two-way AAI between ‘*Candidatus* O. hominis’ and UMN-88 is approximately 62 % based on three quarters of predicted proteins. Another measure, POCP [19], may be used to gauge the relatedness of two genomes at the genus level. To be considered conserved for this measure, a gene must share >40 % amino acid identity over >50 % of its length: two members of the same genus are expected to have at least half of their proteins in common. The POCP between UMN-88 and ‘*Candidatus* O. hominis’ is approximately 58 %. Although these figures are based on draft genomes, as 50.7 % of UMN-88 proteins are conserved in ‘*Candidatus* O. hominis’ from these data, they are likely to be distantly related members of the same genus. The two genomes OH-22767 and OH-22803 have an ANI of 98.78 %, above the 96 % threshold for strains of the same species [30].

**Table 3.** Comparison of '*Candidatus* O. hominis’ draft genomes and *O. rhinotracheale* genome UMN-88

Comparison	ANI		AAI		POCP	
OH-22767 vs UMN-88	81.7 %	149 fragments aligned	61.8 %	75.0 % proteins aligned	57.6 %	50.7 % *O. rhinotracheale* conserved 66.7 % ‘*Candidatus* O. hominis’ conserved
OH-22803 vs UMN-88	77.7 %	119 fragments aligned	61.5 %	77.7 % proteins aligned	58.1 %	50.7 % *O. rhinotracheale* conserved 68.2 % *Candidatus* O. hominis’ conserved
OH-22767 vs OH-22803	98.8 %	7794 fragments aligned	97.8 %	87.5 % proteins aligned	88.5 %	87.8 % OH-22767 conserved 89.1 % OH-22803 conserved

### Metabolic and functional comparison of ‘*Candidatus* O. hominis’ and *O. rhinotracheale*

The functional pathways present in OH-22767, OH-22803 and *O. rhinotracheale* UMN-88 were reconstructed and compared using kegg Mapper [22]. Altogether there are few differences between the species. ‘*Candidatus* O. hominis’ possesses complete modules that are absent from *O. rhinotracheale* for histidine degradation to glutamate, biosynthesis of NAD and tetrahydrofolate, iron transport, and copper processing. Conversely *O. rhinotracheale* can synthesize biotin but no such pathway is present in ‘*Candidatus* O. hominis’. However, this difference may be due to missing data from the ‘*Candidatus* O. hominis’ assemblies as they also appear to lack a compensatory biotin transporter. *O. rhinotracheale* has complete modules for the synthesis of branched chain amino acids (BCAA), pathways that are entirely absent in ‘*Candidatus* O. hominis’. Instead, ‘*Candidatus* O. hominis’ possesses the BCAA transporter gene *brnQ*, enabling it to take up BCAA from the extracellular environment.

### Detection in swab material using fluorescent hybridization

**Fig. 2.**
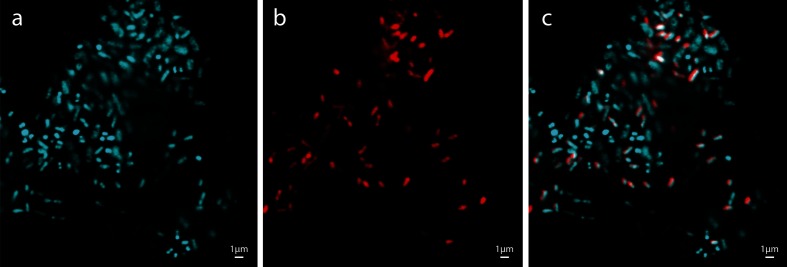
‘*Candidatus* O. hominis’ cells visualized with a fluorescent ribosomal probe. (a) DAPI stain of mixed bacteria from sample 09B00559, (b) ‘*Candidatus* O. hominis’ specific rRNA probe, (c) overlay of (a) and (b). From previous data, the expected microbial profile of the sample is approximately 49 % *Ornithobacterium*, 38 % *Moraxella* taxon I, 4 % *Moraxella* taxon II, 4 % *Streptococcus*, 2 % *Helcococcus*, 2 % *Haemophilus*, and <1 % *Corynebacterium, Dosoligranulum* and *Brachybacterium*.

A fluorescent probe targeting the V5 region of the 16S ribosomal RNA was designed for ‘*Candidatus* O. hominis’ using full-length sequences of the 16S rRNA gene. It was used to visualize ‘*Candidatus* O. hominis’ cells directly from nasopharyngeal swab samples of mixed bacteria (Fig. 2). ‘*Candidatus* O. hominis’ is a Gram negative-bacillus, often observed in pairs and occasionally longer chains, similar to the morphology of *O. rhinotracheale*. *O. rhinotracheale* was tested in parallel and was not bound by the ‘*Candidatus* O. hominis’ probe.

### Core genome

**Fig. 3.**
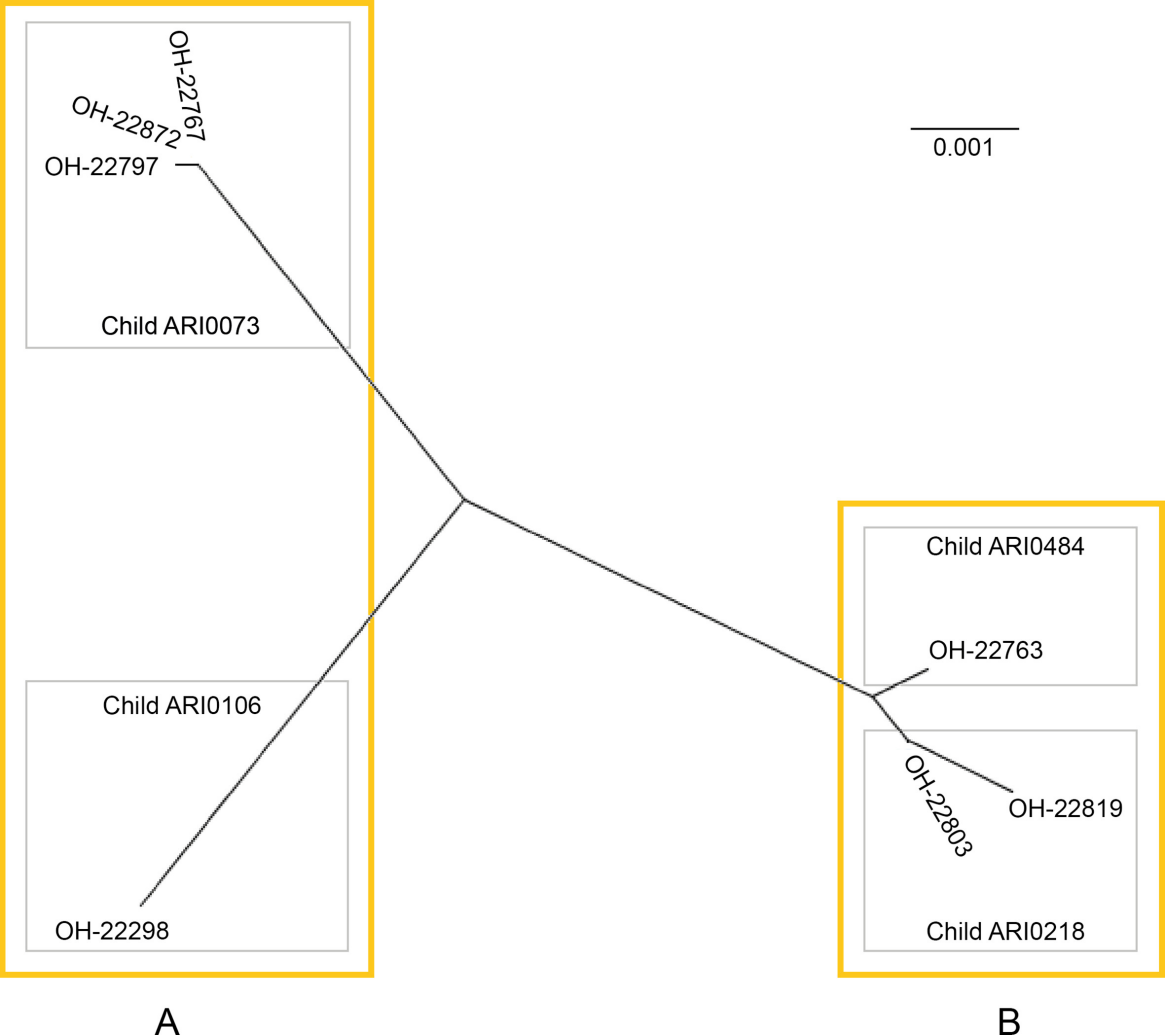
Relationship between ‘*Candidatus* O. hominis’ samples. Unrooted phylogenetic tree built from 935 kb core genome sequence from high- and low-quality assemblies using RAxML. Grey boxes represent samples from individual infants, orange boxes indicate groups based on the accessory genome. Scale bar indicates mean nucleotide substitutions per site.

The core genes shared between the draft genomes and lower quality assemblies, along with the accessory genes unique to each, were calculated using Roary [20]. Due to the lower quality assemblies containing gaps, just 935 kb or approximately 50 % of the draft genome size was identified as ‘core genome’ using this analysis. A core genome phylogeny was generated using >13 000 SNPs identified in this shared sequence [31]. Samples taken from the same child at different dates are very similar (Fig. 3), adding to the initial 16S rRNA gene oligotype data that inferred long-term carriage of the same or closely related strains in this cohort. Features of interest that are present in the core genome include a large toxin gene *toxA* and gliding motility-associated genes.

**Fig. 4.**
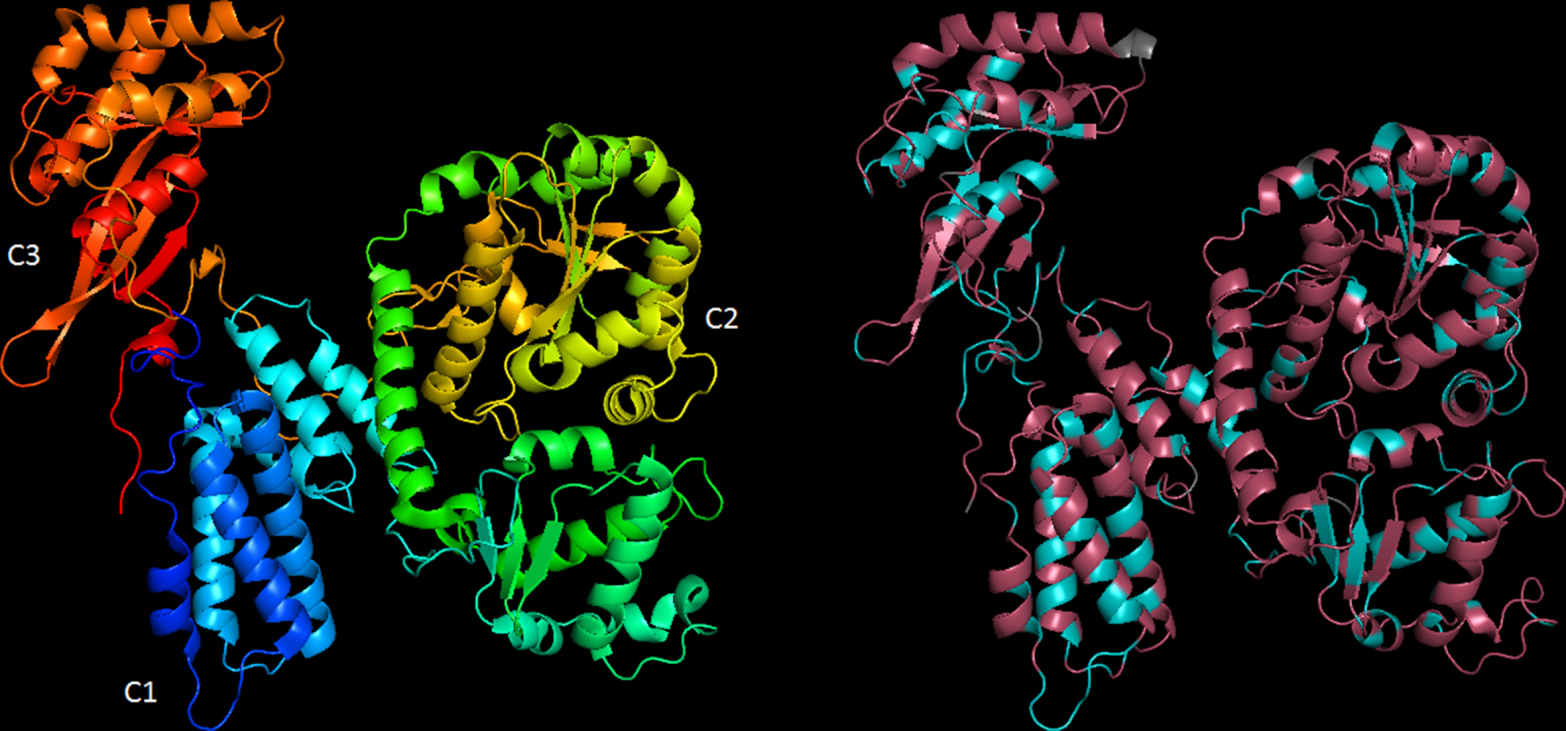
Structure of PMT C-terminal domain, and a model of the equivalent region of ‘*Candidatus* O. hominis’ ToxA. The PMT structure (left) is coloured on a rainbow spectrum to indicate position. The ‘*Candidatus* O. hominis’ model (right) is coloured according to amino acid identity to PMT with identical residues in blue, non-identical in purple and insertions in grey. In PMT, the C1 region is responsible for plasma membrane localization, the C3 region forms an active pocket and is responsible for mitogenic activity in mammalian cells.

The 3.8 kb gene *toxA* is present in all sequenced samples and was detected by PCR in all 16S-positive swabs from children. It is predicted to produce a secreted toxin similar to the *Pasteurella* mitogenic toxin (PMT). Although ‘*Candidatus* O. hominis’ ToxA and PMT share only 35 % amino acid identity overall, there is greater conservation around the predicted active sites, and the modelled structure of the C-terminal domain is extremely similar (Fig. 4).

The core genome includes a full complement of 14 *gld* genes that are homologues of those required for gliding motility by *Flavobacterium johnsoniae*. This mechanism involves the movement of an adhesin around the cell membrane in a helical path, thereby pulling the bacterium rapidly along a substrate [32]. Most of these genes in *Flavobacterium johnsoniae* are also components of the Bacteroidetes type IX secretion system [33, 34], which is responsible for secretion of *Flavobacterium johnsoniae* gliding adhesins SprB and RemA. However, *sprB* has not been identified in ‘*Candidatus* O. hominis’.

*‘Candidatus* O. hominis’ is predicted to have three copies of the rRNA operon. One of these loci assembled in OH-22767 and OH-22803, while two further loci were inferred from mapped read pairs, and the locations confirmed by PCR: 16S rRNA genes are adjacent to OH-22803 [Data Citation 3] contigs D2 and F1, and 23S rRNA genes are adjacent to contigs B1 and E1.

### Accessory genome

Around half of the accessory genome is made up of hypothetical protein genes, most of which are similar to those of other members of the *Flavobacteriaceae* such as *O. rhinotracheale*, *Capnocytophaga, Chryseobacterium, Elizabethkingia, Flavobacterium, Riemerella* or *Weeksella*. It also includes evidence of mobile elements and associated drug-resistance genes, *rhs* (rearrangement hotspot) genes, and two distinct lipopolysaccharide (LPS) production clusters.

A portion of the hypothetical protein genes encode the *Fibrobacter succinogenes* major paralogous domain (PF09603). Up to 17 of these genes are present in each genome. A quarter of them are also predicted to possess an immunoglobulin-like fold (IPR013783). No two samples have a complement of completely identical predicted genes, but more are shared between samples that group together based on the core genome (Fig. 3).

The genomes contain different complements of bacteriophage-associated genes, type I, II and III restriction modification systems, diverse variants of DNA degradation genes *dndCDE* [35], abortive infection systems, and transfer and mobilization genes. In some cases, these are present in small regions of <10 kb and lack any clear structure, while in others they are part of a defined element such as the 30 kb tailed bacteriophage (22803_00899–00942) [Data Citation 3] or the 19 kb element (22803_01685–01710) containing efflux genes and flanked by 350 bp imperfect direct repeats. All sequenced samples possess efflux transporters of the MATE and RND families, but genomes from cluster A (Fig. 3) also have a B1 metallo-β-lactamase (22767_01182) [Data Citation 2], streptogramin lyase (22767_01181) and *ampC* gene (22767_01179) within a partially-assembled mobile element. Genomes from cluster B possess an extended-spectrum β-lactamase gene *per1* on the well-characterized transposon Tn*4555* [36].

The accessory genome of strains from cluster A encodes a number of elements containing the conserved RHS repeat-associated core domain (TIGR03696) with extremely variable C-termini and unique hypothetical protein genes immediately downstream. There is one large *rhs* gene of >9 kb, 22767_01758, that encodes a protein sharing signatures with the *Salmonella* plasmid virulence protein SpvB (IPR003284) and the bacterial insecticide toxin TcdB (IPR022045), while the other smaller ORFs may be the dissociated tips generated from lateral acquisition of variable C-termini as seen in *Serratia marcescens* [37]. This large *rh*s gene with displaced tips is only found in samples from two children, ARI0106 and ARI0073; some but not all of the *rhs* gene tips differ between them. These samples also possess a further *rhs* gene with no displaced tips, that has two predicted phospholipase D domains (IPR001736). Some Rhs proteins have been shown to act as competition factors [38], consistent with ‘*Candidatus* O. hominis’ being a persistent member of the nasopharyngeal microbiota.

**Fig. 5.**
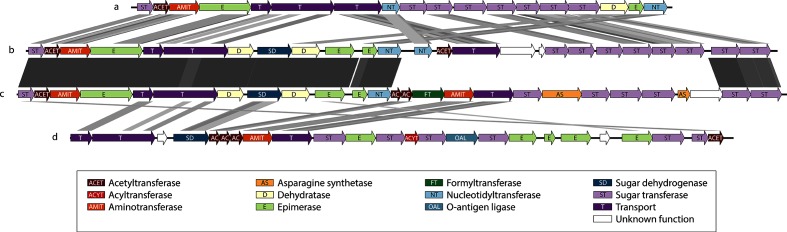
tblastx alignment of ‘*Candidatus* O. hominis’ LPS clusters and those of related species. (a) *O. rhinotracheale* serotype A, type strain DSM15997 (LPS genes Ornrh 0868–0885), (b) ‘*Candidatus* O. hominis’ variant A, OH-22767 (LPS genes 22767_00484–00508), (c) ‘*Candidatus* O. hominis’ variant B, OH-22803 (LPS genes 22803_00489–00514), (d) *Chryseobacterium* sp. YLOS41 (LPS genes DQ356_07110–07225).

Although all the genomes possess a 30 kb LPS production gene cluster (Fig. 5a–d), 11 kb of this region differs between samples from clusters A and B, containing a different set of transferases and synthases, and suggesting the presence of at least two serotypes in the species. The A variant (Fig. 5b) includes an ABC transporter similar to *O. rhinotracheale* serotype A [Data Citation 7], while the B variant (Fig. 5c) has a Wzx flippase similar to *Chryseobacterium* sp. YLOS41 [Data Citation 8].

### Summary description of the provisional species

'*Candidatus* O. hominis’ is a proposed member of the established genus *Ornithobacterium* within the family *Flavobacteriaceae*. It is an uncultured Gram-negative bacillus. It may be identified using the full length 16S rRNA gene sequence (accession numbers LS992475-6 [Data Citation 4 and 5]) or with the 16S rRNA probe 5′-GUUCUUCACCCCGAAAACG-3′. The bacterium inhabits the human nasopharynx and is an aerobic mesothermophile. Samples described here are derived from nasopharyngeal swabs from children under the age of 2 years in Thailand.

## Discussion

Previously there has been little evidence available for ‘*Candidatus* O. hominis’ colonizing adults in Thailand or other countries, as most nasopharyngeal microbiota studies target young children. By screening 100 pairs of mother/child samples (Table 2), 2 % of samples from mothers and 42 % from infants were unequivocally PCR positive for ‘*Candidatus* O. hominis’. The prevalence at 12 months of age could be estimated as 32.3–51.7 % (95 % CI) using this screen, but the maternal prevalence could not be accurately measured from this number of samples. The *C*_t_ values for mothers may indicate a lower bacterial load, leading to a modest under-detection of colonization by PCR. It is in contrast with other bacteria such as *Streptococcus pneumoniae*, which is commonly carried in both adults and infants in Maela: among these 100 pairs of swabs 31 % from mothers and 79 % from infants were culture positive for *Streptococcus pneumoniae*.

Preliminary efforts have been made to culture the bacterium from nasopharyngeal swabs expected to contain a large proportion of this species, following protocols appropriate for *O. rhinotracheale* or other nasopharyngeal bacteria. ‘*Candidatus* O. hominis’ has not yet been successfully cultured from any archived sample. Metabolic comparison of ‘*Candidatus* O. hominis’ and *O. rhinotracheale* suggests that BCAA can be produced by *O. rhinotracheale* but must be imported from the environment by ‘*Candidatus* O. hominis’; however, rich media such as brain heart infusion or blood/chocolate agar should provide adequate amino acids for uptake. There may be other metabolic requirements we have not yet identified that have prevented successful cultivation, or it may be that the cells have not survived storage due to environmental stresses [39].

In earlier work [1], it was noted that each child was colonized with only one of the three detected 16S rRNA gene oligotypes representing ‘*Candidatus* O. hominis’. Colonization was persistent, i.e. constituting >5 % of the proportional abundance of taxa for at least 5 consecutive months in 13 out of 21 children, but was detected in all children at some point during the study. Here, we describe multiple similar ‘*Candidatus* O. hominis’ genomes taken from time-points that were 4–10 months apart in two children, adding evidence to the hypothesis that long-term colonization is restricted to a particular strain for each host. Given the high prevalence of ‘*Candidatus* O. hominis’ in Maela and the genetic diversity observed between contemporaneous samples from only four children, this exclusion of diversity from the host may be explained by microbial competition systems, such as the SdpABC-like toxin system identified on a mobile element in OH-22298 [40] or Rhs proteins present in all samples [38]. Due to the high frequency of clinical pneumonia in Maela (0.73 episodes per child year [8]), the children and their microbiota are frequently exposed to β-lactam antibiotics. In all sequenced samples, we found evidence of horizontally acquired drug-resistance genes, which may also aid persistent colonization.

Bacterial LPS may confer advantages in adhesion and avoidance of complement-mediated cell lysis, although it is also a key target for the host immune system [41]. The gene content of LPS cluster variant A is somewhat similar to that of the *O. rhinotracheale* serotype A (Fig. 5a), the most common of the 18 known serotypes [42] and the only *O. rhinotracheale* LPS type currently represented in public sequence databases. Despite individual gene similarity to LPS clusters of several *Chryseobacterium* species, including *Chryseobacterium gallinarum* and *Chryseobacterium senegalense*, ‘*Candidatus* O. hominis’ variant B in its entirety does not resemble those from any known genomes.

Gliding motility is often associated with firm dry surfaces [43], though it is also found among oral bacteria such as those from the genera *Cytophaga* and *Capnocytophaga*. The ability to move independently in the environment is advantageous for scavenging nutrients, for complex biofilm formation and to bring about contact with other bacterial or host cells. The *gld* genes that are required for gliding motility among the Flavobacteriia, Cytophagia and Sphingobacteriia overlap with those of the type IX secretion system, so a subset of these genes is also found in non-motile relatives [44]. Despite possessing all 14 *gld* genes and further required genes *sprAET*, these ‘*Candidatus* O. hominis’ genomes do not include a SprB-like adhesin and putative gliding motility must be confirmed phenotypically.

PMT is a toxin produced by some serovars of *Pasteurella multocida* that causes a range of host pathologies including nasal bone resorption [45], lower respiratory tract disease [46, 47] and dermonecrotic wound infections [48], and has also been shown experimentally to affect the heart [49], liver [50] and bladder [51]. It acts by deamidating the α subunits of several heterotrimeric G proteins, thereby activating mitogenic signalling pathways [52, 53]. In the Maela cohort, there are no reports of PMT-like toxin mediated disease, strongly suggesting that despite structural similarities the expression or function of ‘*Candidatus* O. hominis’ ToxA is different to that of *P. multocida*.

In conclusion, we have assembled seven genomes representing a new species of nasopharyngeal bacteria proposed as ‘*Candidatus* O. hominis’, from nasopharyngeal swabs collected from a cohort of children in the Maela refugee camp in Thailand. Although phenotypic characterization is not yet possible due to undetermined storage or culture requirements, several points of interest have been identified for further investigation. These include the predicted gliding motility phenotype, two LPS variants and the production of a protein similar to the *Pasteurella* mitogenic toxin. The prevalence of ‘*Candidatus* O. hominis’ colonization appears to be approximately 42 % of 12-month-old children in Maela refugee camp and needs to be estimated in other populations.

## Data bibliography

1. Salter S J, Scott P, Page A J, Tracey A, de Goffau M C, Cormie C, Ochoa-Montaño B, Ling C L, Tangmanakit J, Turner P, Parkhill J. EMBL European Nucleotide Archive (ENA) project accession number ERP107699 (2019).
2. Salter S J, Scott P, Page A J, Tracey A, de Goffau M C, Cormie C, Ochoa-Montaño B, Ling C L, Tangmanakit J, Turner P, Parkhill J. NCBI sequencing project accession number UNSC01000000 (2019).
3. Salter S J, Scott P, Page A J, Tracey A, de Goffau M C, Cormie C, Ochoa-Montaño B, Ling C L, Tangmanakit J, Turner P, Parkhill J. NCBI sequencing project accession number UNSD01000000 (2019).
4. Salter S J, Scott P, Page A J, Tracey A, de Goffau M C, Cormie C, Ochoa-Montaño B, Ling C L, Tangmanakit J, Turner P, Parkhill J. NCBI partial 16S rRNA gene accession number LS992475 (2019).
5. Salter S J, Scott P, Page A J, Tracey A, de Goffau M C, Cormie C, Ochoa-Montaño B, Ling C L, Tangmanakit J, Turner P, Parkhill J. NCBI partial 16S rRNA gene accession number LS992476 (2019).
6. Zehr E S, Bayles D O, Boatwright W D, Tabatabai L B, Register K B. NCBI genome accession number CP006828.1 (2014).
7. Lucas S, Copeland A, Lapidus A, Goodwin L, Pitluck S, Peters L, Mikhailova N, Teshima H, Kyrpides N, Mavromatis K, Pagani I, Ivanova N, Ovchinnikova G, Zeytun A, Detter J C, Han C, Land M, Hauser L, Markowitz V, Cheng J-F, Hugenholtz P, Woyke T, Wu D, Lang E, Kopitz M, Brambilla E, Klenk H-P, Eisen J A.NCBI genome accession number CP003283.1 (2014).
8. Li C-M. NCBI sequencing project accession number QPIE00000000.1 (2018).
